# Rap1A Regulates Osteoblastic Differentiation via the ERK and p38 Mediated Signaling

**DOI:** 10.1371/journal.pone.0143777

**Published:** 2015-11-24

**Authors:** Yougen Wu, Juan Zhou, Yinghua Li, Yunjiao Zhou, Yunqing Cui, Gong Yang, Yang Hong

**Affiliations:** 1 Central Laboratory, The Fifth People's Hospital of Shanghai, Fudan University, Shanghai 200240, China; 2 Cancer Institute, Fudan University Shanghai Cancer Center, Shanghai, 200032, China; 3 Department of Oncology, Fudan University Shanghai Medical College, Shanghai, 200032, China; 4 Department of Osteology, The Fifth People’s Hospital of Shanghai, Fudan University, Shanghai 200240, China; University of Texas Southwestern Medical Center, UNITED STATES

## Abstract

Rap1A is a member of small G proteins belonging to the Ras family. Recently, an integration of human genome-wide association studies (GWAS) and gene expression profiling study revealed that single-nucleotide polymorphisms (SNPs) within human Rap1A were strongly associated with narrow neck width in women. However, the regulatory role of Rap1A in osteoblasts remains to be elucidated. Here we report that Rap1A is a key regulator in osteoblast differentiation. Rap1A expression and activity were gradually enhanced during the induced differentiation of multipotent mesenchymal progenitor cells (C2C12) and preosteoblastic cells (MC3T3-E1). Knockdown of endogenous Rap1A significantly inhibited the osteogenic marker gene expression and matrix mineralization in cells with osteogenesis. In addition, knockdown of endogenous Rap1A suppressed the activation of extracellular signal-regulated kinase (ERK) and p38 mitogen-activated protein kinase (MAPK), while overexpression of Rap1A accelerated osteoblast differentiation and enhanced the phosphorylation of ERK and p38. Taken together, our study suggests that Rap1A regulates osteoblast differentiation through modulating the ERK/p38 signaling.

## Introduction

Osteoblast proliferation, differentiation and maturation are crucial events in new bone formation and bone quality [1]. Osteoblast differentiation is a complicated process including the commitment of pre-osteoblasts to matrix-secreting osteoblasts. Osteoblasts synthesize and secrete a variety of bone-specific marker proteins including the runt-related transcription factor 2 (Runx2), alkaline phosphatase (Alp), the collagen type I alpha 1 (Col1a1), osteocalcin (OCN), osteopontin (OPN), osterix and bone sialoprotein (BSP), which not only act as structural supports, but also play vital roles in osteoblast maturation and biological function including cell interaction, communication, matrix deposition, and mineralization [2, 3].

Bone morphogenetic protein-2 (BMP2) has been widely studied and is known to have a strong osteogenic capacity both in vitro and in vivo [4]. BMP2 exerts its effects through serine/threonine kinase receptors to induce phosphorylation of Smad 1/5/8, while Smad 1/5/8 binds Smad 4 to form a complex that is transported to the nucleus to regulate transcription of target genes, such as Runx2 [5, 6]. In addition to the canonical BMP receptor–Smad pathway, BMPs can elicit the non-canonical MAPK pathway. BMPs have been reported to activate the MAPK/ERK/p38/JNK signal pathway. Numerous studies have demonstrated that the MAPK/ERK/p38/JNK pathway regulates both in vitro osteoblast differentiation and in vivo bone formation [7–9].

Rap1A is a member of small G proteins belonging to the Ras oncogene family functioning in numerous biological processes including cell proliferation, adhesion, spreading, migration and cancer progression [10]. Like other small G proteins, rap1A functions as a molecular switch through alternating between an active GTP-bound and an inactive GDP-bound state [11]. Recently, an integration of human GWAS and gene expression profiling study revealed that SNPs within human Rap1A were strongly associated with narrow neck width in women [12], indicating that Rap1 may have an essential role in regulating osteoclast function. A study has shown that Rap1 deletion in mature osteoclasts retarded pathological bone loss [13]. However, the exact role and the underlying mechanism mediated by Rap1A to regulate osteoblastic differentiation are unclear.

In the present study, we examined the role of Rap1A in the multipotent mesenchymal progenitor cell line C2C12 and the osteoblastic cell line MC3T3-E1. We show that knockdown of Rap1A inhibited the expression of bone related genes, resulting in the inhibition of osteoblast differentiation. Overexpression of Rap1A enhanced osteoblast differentiation in MC3T3-E1 cells. These results suggest that Rap1A is an important regulator of osteoblast differentiation.

## Materials and Methods

### Reagents and Antibodies

Ascorbic acid (AA), β**-**glycerophosphate (β**-**GP), Cetylpyridinium Chloride, and Alizarin Red S were purchased from Sigma Aldrich. The recombinant human bone morphogenetic protein-2 (rhBMP-2) was obtained from R&D Systems. MEK1/2 inhibitor U0126 and p38 inhibitor SB203580 were obtained from Selleck, Shanghai, China. Antibody to Rap1A was from Santa Cruz Biotech. Antibodies to p-ERK1/2, ERK1/2, pp38, p38, HA, and β-actin were purchased from Cell Signaling Inc.

### Plasmids

The lentivirus system for short hairpin RNA (shRNA) expression including three plasmids (pLKO.1-puro, psPAX2, and pMD2.G) was obtained from Addgene (Cambridge, MA, USA). The lentiviral vector pCDH-CMV-MCS-EF1-Puro was obtained from Addgene System Biosciences (SBI). To knockdown of Rap1A, two Rap1A shRNAs (Rap1A shRNA-1, 5’GCTCAGTCTACGTTTAATGAT3’; Rap1A shRNA-2, 5’CCGAGCAATTTACAGCAATGA3’) targeting mouse Rap1A sequences were annealed and inserted into the lentiviral vector pLKO.1-puro. Full-length coding DNA sequence of mouse Rap1A was amplified using the forward primer 5’-GCGGAATTCatgcgtgagtacaagctagt -3’ and the reverse primer with HA tag: 5’-GCGGGATCCctaAGCGTAATCTGGAACATCGTATGGGTAgagcagcaaacatgatttcttt-3’.The PCR product was cloned into the lentiviral vector pCDH-CMV-MCS-EF1-Puro.

### Lentivirus production and transduction

To generate lentiviruses, lentiviral vector (pLKO.1-puro or pCDH-CMV-MCS-EF1-Puro), psPAX2, and pMD2.G were co-transfected into HEK293T cells using Lipofectamine^2000^ (Invitrogen). Eight hours after transfection, the medium was changed. Supernatants were collected 48 hours after transfection and filtered through a 0.45-μm membrane (Millipore). Target cells were infected with virus in the presence of 8 μg/ml polybrene (Sigma) for 12 to 24 hours. To get stable Rap1A knockdown and overexpressing cells, growth medium containing 3 μg/mL of puromycin (Sigma) was added to select cells for 1 week before used for next experiments.

### Cell culture and osteogenic induction

MC3T3-E1 cell line, kindly gifted from Dr. Yi Lu (Center for Translational Medicine, Guangxi Medical University, Nanning, Guangxi, China), was originally purchased from ATCC (Manassas, VA). MC3T3-E1 cells were cultured in α-MEM medium (no ascorbic acid) (Invitrogen, Carlsbad, CA) with 10% FBS and 1% penicillin/streptomycin (Invitrogen). C2C12 cell line was got from Dr. Mingyao Liu (Shanghai Key Laboratory of Regulatory Biology, Shanghai, China) and cultured in DMEM with 10% FBS [14]. 293T cells (American Type Culture Collection, Manassas, VA) were grown in DMEM with 10% FBS. Osteoblast differentiation was induced with osteogenic medium containing 50 μg/ml ascorbic acid and 10 mM β-glycerophosphate (osteogenic culture conditions) with or without 100 ng/ml BMP2 and culture medium was replaced every 3 d.

### MTT assay

C2C12 cells or MC3T3-E1 cells were cultured at 3×10^3^ cells/well in a 96-well plate in triplicate. Subsequently, the cells were cultured for 4 days. 5 mg/mL MTT solution (Sigma) was added (1 part to 10 parts medium) to each well and the plate was incubated at 37°C for 4 h. Then, the supernatant was removed and dimethyl sulfoxide was added to each well to dissolve the formazan crystals. The OD at 490 nm was quantified using a Tecan Infinity 200PRO multi-well plate reader (Tecan Ltd., Switzerland).

### Alkaline phosphatase staining

Cells were rinsed in PBS, fixed in 4% paraformaldehyde for 20 minutes at room temperature, washed with PBS, and then stained with One-Step NBT-BCIP (catalog no. 34042; Thermo Scientific) for 20 minutes, rinsed with PBS, and air dried.

### Alkaline phosphatase activity assay

ALP activity assay was determined using a commercial kit (Nanjing Jiancheng Bioengineering Institute, Nanjing, China) according to the manufacturer’s instructions. A bicinchoninic acid (BCA) method (Pierce, Rockford, IL, USA) was performed to detect the protein concentration. The relative ALP activity was normalized to total protein concentration.

### Alizarin red S (ARS) staining

For alizarin red staining, cells were fixed with 70% ethanol, and rinsed three times with ddH_2_O to remove ethanol completely. The cells were then stained with 40mM alizarin red stain (AR-S) solution (pH 4.2) for 15 minutes to label the calcium deposits. After that, cells were rinsed with ddH_2_O five times to remove unbound AR-S. The stained cultures were photographed. To quantify the AR-S stain, the AR-S was dissolved with 10% (w/v) cetylpyridinium chloride in 10mM sodium phosphate (pH 7.0). The extracted solution was measured by using a spectrophotometer at 562 nm.

### Rap1A activity assay

Rap1A activity assay was determined using a commercial kit (catalog no. 17–321; Millipore) according to the manufacturer’s instructions. In brief, cell lysates were prepared with Rap1 activation lysis buffer (50 mM Tris-HCl, pH 7.4, 0.5 M NaCl, 1% NP40, 2.5 mM MgCl2, and 10% glycerol, with proteinase inhibitor cocktail) and incubated with a GST-tagged fusion protein, corresponding to residues 788–884 of human Ral GDS-Rap binding domain (GST-RalGDS-RBD), immobilized on glutathione-agarose at 4°C for 45 minutes. The amount of GTP-bound Rap1A (from the pulldown) and total Rap1A (from a sample of reserved total cell lysate) was determined by Western blot analysis using an anti-Rap1A antibody.

### Quantitative Real-Time RT-PCR Analysis

Total RNA was extracted from cells using TRIZOL reagent (Invitrogen; Carlsbad, CA). 2 μg of total RNA was reversely transcribed into cDNA by using SuperScript® First-Strand Synthesis System (Invitrogen™). The cDNA samples were subjected to PCR analysis using faststart universal SYBR green master rox (Roche). Amplifications were performed on an ABI 7500 Real-time PCR system. The optimal conditions were defined as follows: 95°C for 10 min, followed by 40 cycles at 95°C for 15 s and at 60°C for 1 min, and melting curve analysis at 95°C for 15 s, at 60°C for 1 min, at 95°C for 15 s, and at 60°C for 15 s. The relative mRNA expression of the marker genes of osteoblastic differentiation was adjusted according to the expression of GAPDH. The primer pairs used for PCR were as follows: Rap1A, forward 5’- TATGACCCAACGATAGAAG-3’, reverse 5’- CATTAAACGTAGACTGAGCT-3’; Runx2, forward 5’-GCCGGGAATGATGAGAACTA-3’, reverse 5’-GGACCGTCCACTGTCACTTT-3’; Col1a1, forward 5’- GCTCCTCTTAGGGGCCACT-3’, reverse 5’- CCACGTCTCACCATTGGGG-3’; Osteocalcin, forward 5’-TGCTTGTGACGAGCTATCAG-3’, reverse 5’-GAGGACAGGGAGGATCAAGT-3’; Osterix, forward 5’- GTCAAGAGTCTTAGCCAAACTC-3’, reverse 5’- AAATGATGTGAGGCCAGATGG-3’; And GAPDH, forward 5’-TGAACGGGAAGCTCACTGG-3’, reverse 5’-TCCACCACCCTGTTGCTGTA-3’. The results were analyzed and validated using the relative standard curve method and the delta-delta Ct method.

### Western blot analysis

Cells were lysated with radioimmunoprecipitation buffer (Beyotime, Shanghai, China) supplemented with proteinase inhibitor cocktail (Roche Applied Science). Equivalent amounts of protein measured with BCA protein Assay Kit were denatured in SDS sample buffer, separated on polyacrylamide-SDS gels and transferred to PVDF membranes. After blocking in Tris-buffered saline containing 5% BSA and 0.1% Tween-20, membranes were incubated with primary antibodies overnight at 4°C. Detection was performed using goat anti-mouse or goat anti-rabbit IgG-horseradish peroxidase secondary antibody for 1 h at room temperature and an ECL detection kit (Immobilon™).

### Statistical analysis

Statistical comparison between the control group and the treatment group was performed using the standard two-tailed Student's t-test. Data were presented as mean ± standard error and a minimum of three independent experiments were performed for each assay. Statistical significance was indicated with * for P < 0.05 or ** for P < 0.01.

## Results

### Osteoblast differentiation induces Rap1A expression

**Fig 1:**
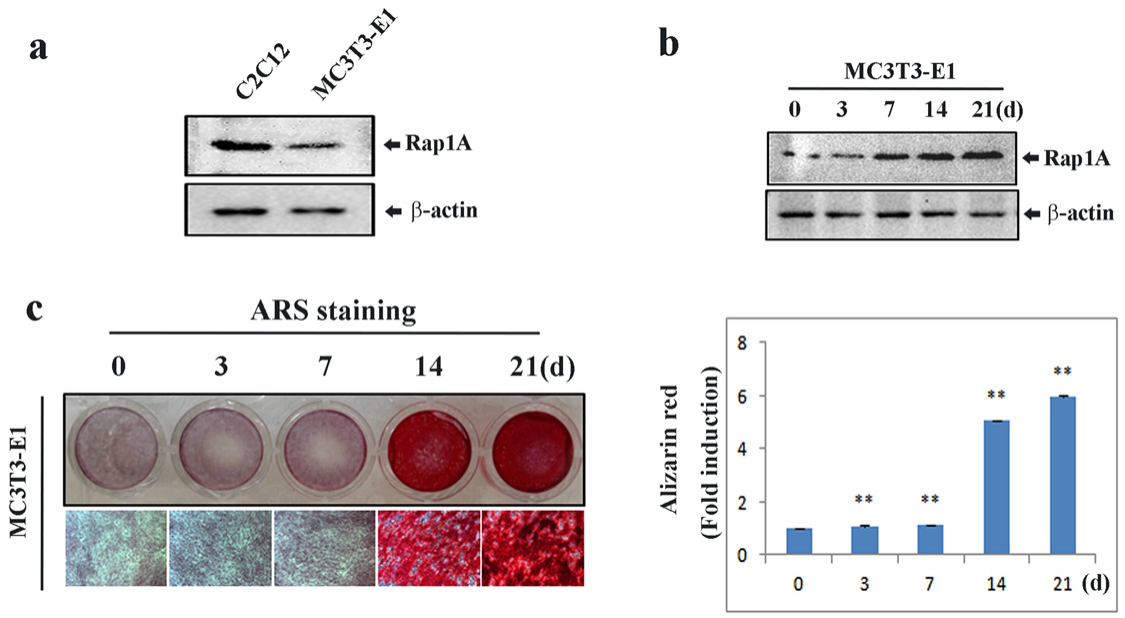
Expression of Rap1A during osteoblastic differentiation. **(a)** Endogenous expression of Rap-1A in MC3T3-E1 and C2C12 cells by Western blotting. **(b)** The temporal change of Rap1A expression in MC3T3-E1 cells during osteoblastic differentiation by Western blotting. **(c)** Alizarin red staining for bone nodules in MC3T3-E1 cells. Original magnification was ×20 (left). Arizalin red-S staining activity was quantified by densitometry at 562 nm (right). Data represent means ± SD of triplicate samples. *P < 0.05, **P < 0.01 vs. the undifferentiated cells. β-actin was used as the internal control.

Before evaluating the effect of Rap1A on osteoblastic differentiation, we examined the endogenous Rap1A expression in C2C12 cells and preosteoblastic MC3T3-E1 cells using Western blotting. We found that Rap1A was higher in C2C12 cells than in MC3T3-E1 cells (**Fig 1A**). We then examined the expression pattern of Rap1A in MC3T3-E1 cells treated with ascorbic acid (AA) and β**-**glycerophosphate (β**-**GP) for osteoblast differentiation. As shown in **Fig 1B and 1C**, Rap1A expression and bone mineralization were gradually increased in a time course at 0, 3, 7, 14, 21 d during osteoblast differentiation.

**Fig 2:**
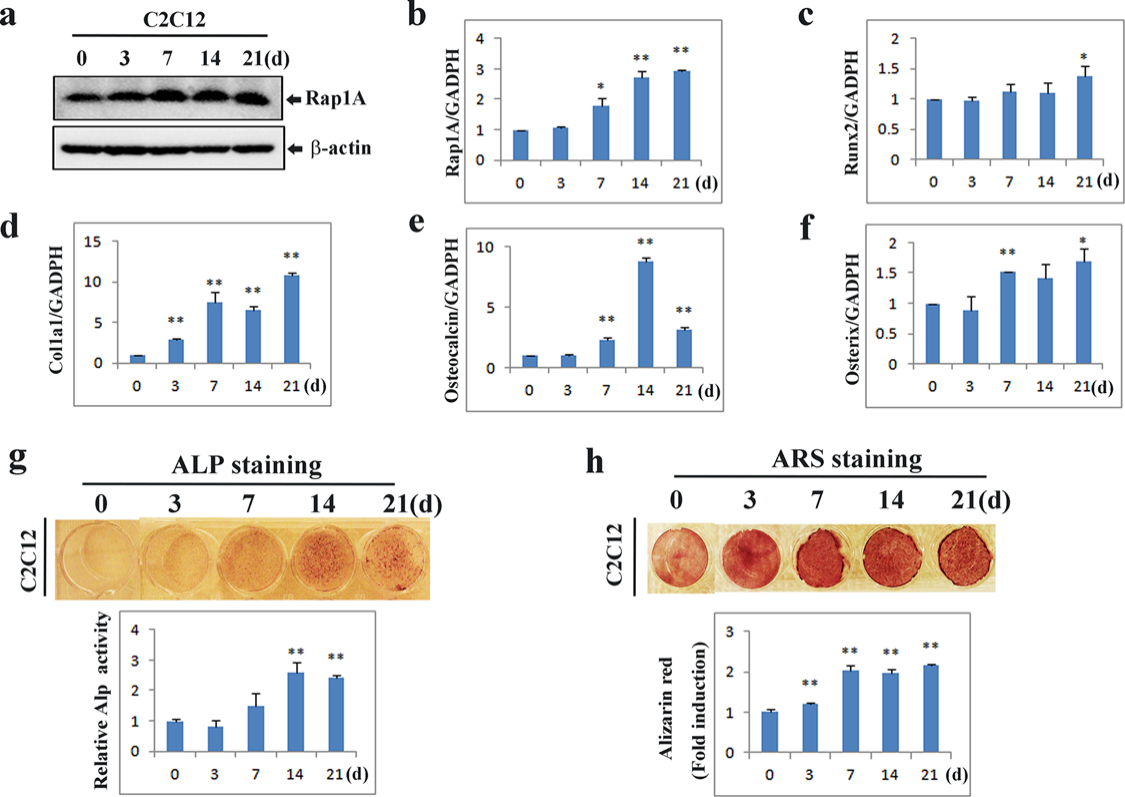
Association of Rap1A with osteoblast differentiation. **(a)** Immunoblot analysis of Rap1A expression during osteogenic differentiation. The protein extracts were immunoblotted with anti-Rap1A antibody. β-Actin was used as the internal control. **(b-f)** Changes of osteoblastic markers gene expression in differentiated C2C12 cells. C2C12 cells were cultured with osteogenic medium containing ascorbic acid (50μg/ml), and β-glycerophosphate (10 mM) for the indicated times. Quantitative real-time PCR was performed for the mRNA expression of Rap1A, Runx2, Col1a1, Osteocalcin, and Osterix. **(g)** Alkaline phosphatase (ALP) staining and ALP activity measurement were determined during osteoblastic differentiation from C2C12 cells. **(h)** Osteoblastic mineralization of C2C12 cells was determined by Alizarin red staining and calcium content quantified in the cellular matrix by spectrophotometer (OD562nm). Data represent means ± SD of triplicate samples. *P<0.05, **P<0.01 vs. the undifferentiated cells.

We next treated C2C12 cells with osteogenic factors containing ascorbic acid (AA) and β**-**glycerophosphate (β**-**GP) and found that the expression of Rap1A was increased along with enhanced differentiation (**Fig 2A and 2B**). Similarly, ALP staining signal, Alizarin Red S staining as well as the expression of classic osteoblast marker genes Runx2, collagen, type I, alpha (Col1a1), osteocalcin, and osterix were increased as the cells were increasingly differentiated (**Fig 2C–2H**). All of these findings suggest that Rap1A may be essential for osteoblast differentiation and bone mineralization.

### Osteogenic stimulation induces Rap1A activation

**Fig 3:**
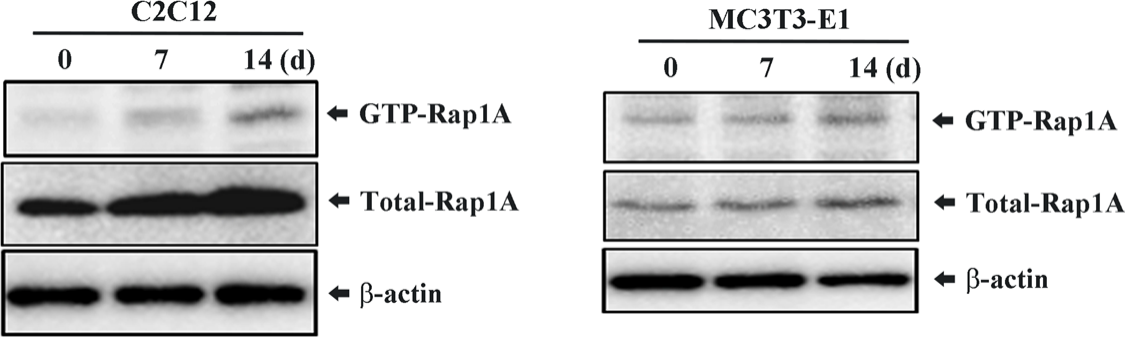
RalGDS pull-downs to assess Rap1A activity induced by osteogenic stimulation. C2C12 and MC3T3-E1 cells were treated with osteogenic inducers (AA/β-GP) for the indicated times and subjected to GST-RalGDS pulldown assays to detect the active form of Rap1A. The levels of captured GTP-Rap1A were determined by immunoblot analysis using an anti-Rap1A antibody. Levels of total Rap1A and β-Actin (loading control) in whole-cell lysates were also determined by immunoblot analysis.

To investigate whether Rap1A is functionally activated during osteoblastogenesis, we examined the changes of the GTP-Rap1A, an activated form of Rap1A, after osteogenic stimulation. As shown in Fig 3, both the levels of the active GTP-bound Rap1A and total Rap1A were increased by treatment with osteogenic inducers (AA/β-GP), indicating that Rap1A can be activated by osteogenic stimulation.

### Knockdown of Rap1A inhibits osteoblast differentiation and osteoblast-specific gene expression

**Fig 4:**
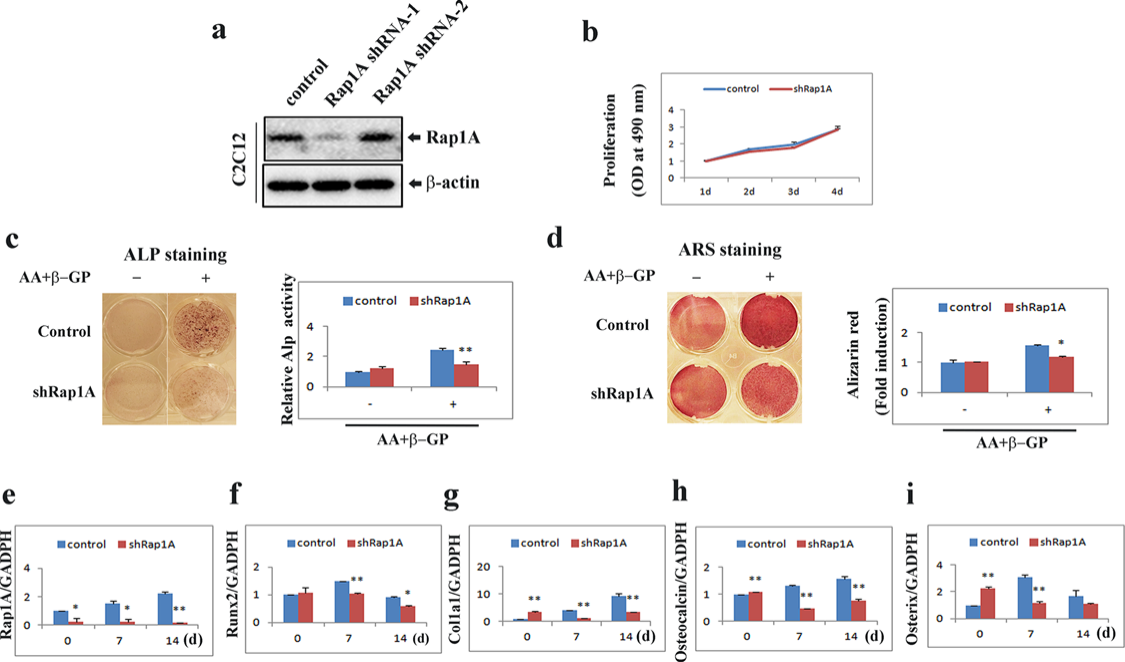
Knockdown of Rap1A inhibits osteoblast differentiation and osteogenic gene expression. **(a)** Representative clones expressing shRNA against Rap-1A were analyzed for Rap1A expression by immunoblot. β-actin was used as a loading control. **(b)** Proliferation of C2C12 cells with or without shRap1A. Relative absorbance of cells in MTT assay was determined at daily intervals over 4 days' incubation. **(c)** Knockdown of Rap1A in C2C12 cells reduced ALP activity. Cells cultured for 10 d with or without osteogenic inducers (AA/β-GP) were fixed and stained for ALP. ALP activities were measured by densitometry at 520 nm (below). **(d)** Knockdown of Rap1A in C2C12 cells reduced mineralized matrix formation. Cells cultured for 14 d with or without osteogenic inducers (AA/β-GP) were fixed and stained for Alizarin red and photographed. The eluted alizarin red stain was quantified by densitometry at 562 nm (below). **(e-i)** Knockdown of Rap1A expression in C2C12 cells suppressed osteoblast-specific gene expression. Transduced cells were cultured with osteogenic medium containing ascorbic acid (50 μg/ml), and β-glycerophosphate (10 mM) for the indicated times and quantitative real-time PCR analysis of mRNA expression of Rap1A, Runx2, Col1a1, Osteocalcin, and Osterix was performed. **(b-i)** Data represent means ± SD of triplicate samples. *, P < 0.05; **, P < 0.01 versus control.

To investigate the potential role of Rap1A in osteoblast differentiation, we silenced the expression of endogenous Rap1A by lentivirus-mediated infection with two specific shRNAs (shRNA1-2). As shown in **Fig 4A**, the Rap1A protein level was significantly reduced in C2C12 cells expressing Rap1A shRNA-1.

We examined the effect of Rap1A on the proliferation of osteoblasts. The proliferation rate of Rap1A shRNA treated C2C12 cells and control cells was not significantly different (Fig 4B). We next examined the effect of Rap1A on osteoblast differentiation and mineralization. The control shRNA and Rap1A shRNA treated C2C12 cells were cultured in the presence or absence of the osteogenic factors. ALP staining and activity were significantly inhibited in Rap1A shRNA treated C2C12 cells compared with control cells (**Fig 4C**). Also, similar to the ALP results, knockdown of Rap1A impaired the formation of mineralized nodules and alizarin red activity under the osteogenic conditions (**Fig 4D**).

We also examined osteoblast-specific gene expression in Rap1A silenced cells. The Real-time PCR results showed that silencing of Rap1A strongly downregulated Runx2, col1a1, osteocalcin and osterix mRNA levels (**Fig 4E–4I**). Collectively, these findings suggest that Rap1A positively regulates osteoblastic differentiation and maturation.

### Silencing of Rap1A decreases ERK and p38 activation

**Fig 5:**
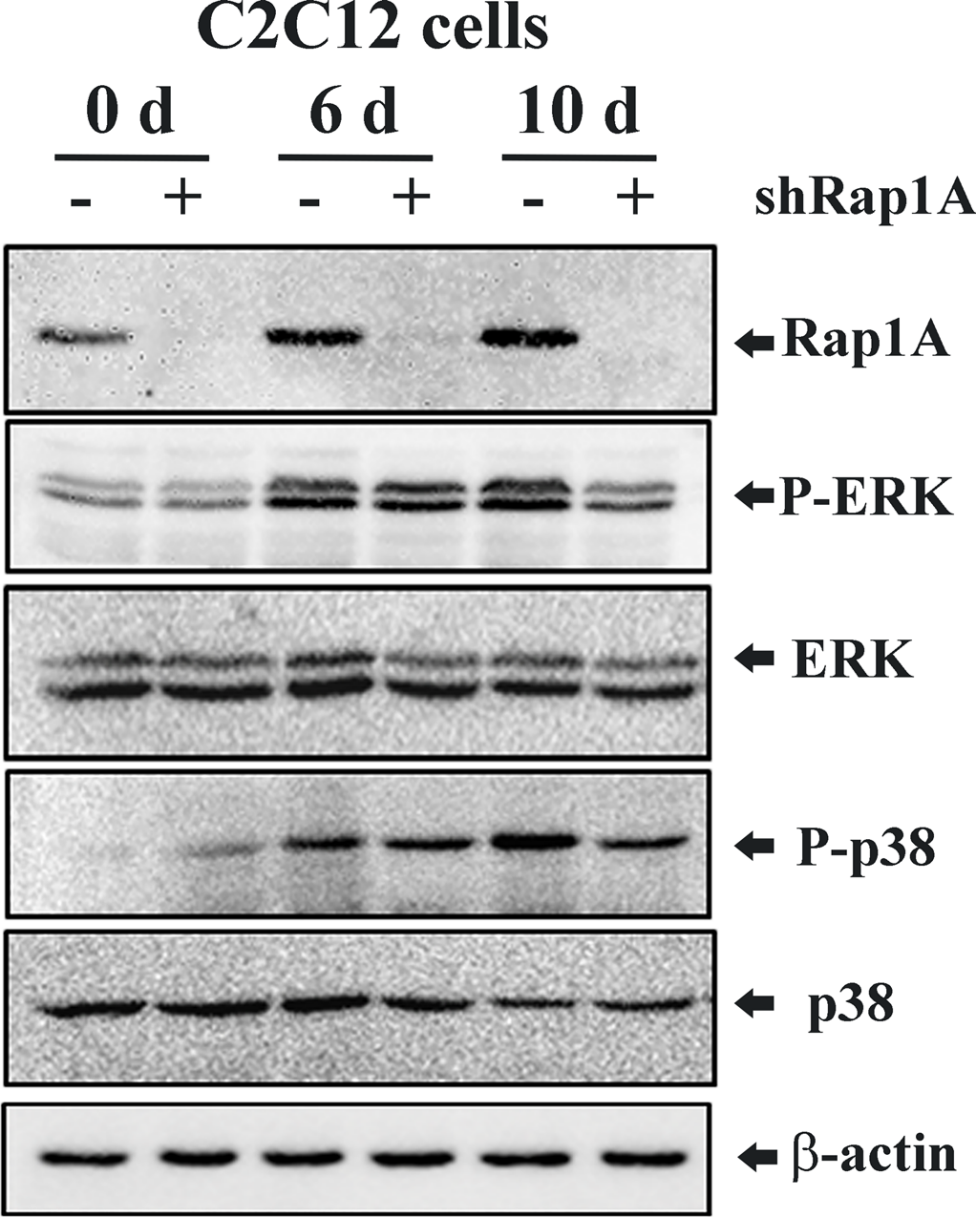
Silencing of Rap1A decreases ERK and p38 activation. Representative immunoblots for Rap1A expression and MAPK activation in control and Rap1A shRNA expressing C2C12 cells grown in basal or osteogenic medium containing ascorbic acid (50 μg/ml), and β-glycerophosphate (10 mM) for 6 and 10 d. Western blot analyses with antibodies recognizing Rap1A, phospho-ERK1/2, total ERK1/2, phospho-p38 and total p38 were performed. β-actin was used for internal control.

To further investigate the mechanism involved in the regulation of osteoblastic differentiation by Rap1A, we examined the MAPK signal pathway in response to osteogenic stimulation in control shRNA and Rap1A shRNA expressing C2C12 cells. As shown in **Fig 5**, the expression of Rap1A protein and phosphorylated ERK1/2 and p38 were markedly enhanced in control shRNA expressing cells undergoing osteogenic differentiation at 10 d, whereas ERK and p38 phosphorylation in Rap1A shRNA transduced cells were blunted compared with control cells under osteogenic induction. Thus, we hypothesized that the MAPK/ERK/p38 cascade may mainly participate in the Rap1A-regulated osteogenic differentiation.

### Overexpression of Rap1A accelerates osteoblast differentiation

**Fig 6:**
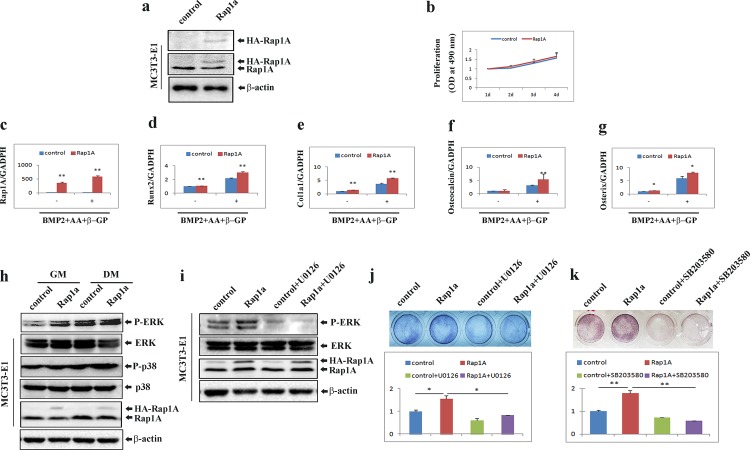
Forced expression of Rap1A accelerates osteoblast differentiation through activation of ERK1/2 and p38 MAPK. **(a)** MC3T3-E1 cells stably expressing Rap1A were established as stated in the ‘‘Materials and Methods,” and Western blotting was used to test Rap1A expression with antibodies recognizing HA and Rap1A. **(b)** Proliferation of MC3T3-E1 cells with or without Rap1A overexpression tested by MTT assays. **(c-g)** Over-expression of Rap1A in MC3T3-E1 cells increased osteoblast-specific gene expression. **(h)** Western blot analyses with antibodies recognizing phospho-ERK1/2, total ERK1/2, phospho-p38, total p38 and Rap1A were performed in cells treated with either growth medium (GM) or osteoblast differentiation medium (DM) containing rhBMP-2 (100 ng/ml), ascorbic acid (50 μg/ml), and β-glycerophosphate (10 mM) for 3 days. β-actin was used as a loading control. **(i)** phospho-ERK1/2, total ERK1/2 and Rap1A were detected by Western blot in MC3T3-E1 cells with or without Rap1A overexpression after treatment with osteoblast differentiation medium (DM) for 3 days in the presence or absence of 10 μM U0126. β-actin was used as a loading control. **(j and k)** Inhibition of ALP activity by U0126 and SB203580 in cells with or without Rap1A overexpression after differentiation induction by osteogenic medium containing rhBMP-2 (100 ng/ml), ascorbic acid (50 μg/ml), and β-glycerophosphate (10 mM) for 3 days in the presence or absence of 10 μM U0126 **(j)** or 10 μM SB203580 **(k)**. Cells were fixed and stained for ALP. ALP activities were measured by densitometry at 520 nm (below). Data represent means ± SD of triplicate samples. *, P < 0.05; **, P < 0.01.

To validate above results, we tested whether Rap1A overexpression promoted osteogenesis. MC3T3-E1 cells stably overexpressing Rap1A were established (**Fig 6A**) and subjected to cell proliferation and differentiation assay. As shown in **Fig 6B**, the proliferation of stable Rap1A overexpression cells was similar to that of control cells. Quantitative real-time PCR analysis showed that Rap1A overexpression cells exhibited significantly increased mRNA expression of Runx2, col1a1, osteocalcin and osterix compared with that in control osteoblasts under osteogenic conditions (**Fig 6C–6G**). Overexpression of Rap1A enhanced the activation of ERK and p38 under osteogenic conditions (**Fig 6H**). At the same time, Rap1A-enhanced activation of ERK was attenuated in cells pretreated with U0126, an inhibitor of MEK1/2 (**Fig 6I**). Moreover, the enforced overexpression of Rap1A significantly increased ALP staining and activity. Treatment of cells with U0126 or the p38-specific inhibitor SB203580 inhibited the ALP activation in Rap1A-overexpressing cells (**Fig 6J and 6K**). These results indicate that Rap1A really stimulates the phosphorylation of ERK and p38 to regulate the differentiation of osteoblast-like cells.

## Discussion

An eSNP (rs494453) located in intron 2 of the Rap1A gene has been reported to be significantly associated with narrow neck width in women [12]. The importance of the present findings is that Rap1A is functionally expressed in multipotent C2C12 and MC3T3-E1 cell lines and appears to promote osteoblastic differentiation and maturation. Although C2C12 cells are more primitive than MC3T3-E1 cells, C2C12 cells are known to differentiate into osteoblast cells in differentiation medium in a similar manner to MC3T3-E1 cells [15, 16]. Interestingly, we examined dynamic changes in Rap1A expression and activation during osteogenic differentiation and found that both the levels of active GTP-bound Rap1A and total Rap1A were increased during osteoblastic differentiation, indicating that Rap1A may contribute to the differentiation of these cell lines.

The activation of the mitogen-activated protein kinases (MAPKs) extracellular signal–regulated kinase (ERK), p38 and c-Jun NH2-terminal kinase (JNK) regulates in vitro osteoblastic differentiation and in vivo bone formation [17–19]. In the non-canonical MAPK pathways, BMP2 activates the ERK1/2, p38, and JNK1/2 mitogen-activated protein kinases to promote osteoblastic differentiation. Numerous studies have demonstrated that inhibition of ERK1/2 or p38 MAPK kinase suppresses osteoblast-specific gene expression [17, 20], whereas inhibition of JNK activity and expression enhances BMP2-induced osteoblastic differentiation [21]. Rap1 has been reported to interact with B-Raf, leading to activation of the ERK/MAPK signaling cascade [22, 23], which contribute to cell migration and inhibit myogenic differentiation [24, 25]. In this study, we found that knockdown Rap1A by shRNA resulted in highly decreased phosphorylation of ERK and p38, which is consistent with earlier studies, suggesting that down-regulation of Rap1A may block the sustained activation of ERK1/2 [26]. Therefore, loss-of-function of Rap1A in osteoblasts may directly impact osteoblast differentiation through the down-regulation of osteoblast marker genes such as Runx2, collagen type I, osteocalcin, and osterix. We also observed that overexpression of Rap1A stimulated the mRNA expression of a panel of osteoblast marker genes and promoted osteoblast differentiation through activation of ERK and p38. Inhibition of either p38 or ERK decreased the effect of Rap1A on ALP regulation, suggesting that the cooperation of ERK1/2 and p38 may play a crucial role in Rap1A-mediated cell maturation and differentiation in osteoblasts. Although MAPK activation is commonly induces cell proliferation, while the up-regulation or down regulation of MAPK including pERK1/2 and pp38 by overexpression or silencing of Rap1A did not alter cellular proliferation of osteoblasts without osteogenic stimulation in the present study.

In conclusion, a major finding of this study is that Rap1A, as a novel positive regulator of osteoblast differentiation and maturation in vitro, regulates osteoblast differentiation, at least in partial, through the ERK and p38 mediated signaling. These data may help to develop new therapeutic strategies through modification of osteoblastic differentiation to promote bone formation. Therefore, Rap1A may be a useful target to develop bone anabolic therapeutics.
